# Risk-Aversion for Negative Health Outcomes May Promote Individual Compliance to Containment Measures in Covid-19 Pandemic

**DOI:** 10.3389/fpsyg.2021.666454

**Published:** 2021-06-18

**Authors:** Chiara Cerami, Caterina Galandra, Gaia Chiara Santi, Alessandra Dodich, Stefano Francesco Cappa, Tomaso Vecchi, Chiara Crespi

**Affiliations:** ^1^Scuola Universitaria Superiore IUSS Pavia, Pavia, Italy; ^2^Cognitive Computational Neuroscience Research Unit, IRCCS Mondino Foundation, Pavia, Italy; ^3^Neurogenetic Research Center, IRCCS Mondino Foundation, Pavia, Italy; ^4^Center for Neurocognitive Rehabilitation, Centro Interdipartimentale Mente/Cervello, University of Trento, Mattarello, Italy; ^5^Dementia Research Center, IRCCS Mondino Foundation, Pavia, Italy; ^6^Department of Brain and Behavioral Sciences, University of Pavia, Pavia, Italy; ^7^Cognitive Psychology Center, IRCCS Mondino Foundation, Pavia, Italy

**Keywords:** COVID-19, risk aversion, negative health outcomes, coping styles, loneliness, empathy

## Abstract

First-person experience of stressful life events can change individuals' risk attitudes, driving to increased or decreased risk perception. This shift to more risk-averse or risk-loving behaviors may find a correlate in the individual psycho-socio-emotional profile. To this purpose, we aimed to estimate the relationship between differences in risk-taking attitudes toward possible negative health outcomes and psycho-socio-emotional dimensions modulating the experience of life-threatening situations, in the context of the Covid-19 pandemic. In March 2020, we launched the PsyCovid Study (https://wprn.org/item/428452) to assess psycho-socio-emotional changes due to Covid-19 pandemic in the Italian population. Additionally, we distributed to 130 participants the Covid-19 Risk Task, including monetary and health-related stimuli, estimating a measure of risk-aversion toward health and classifying participants on the basis of their risk-attitude profiles. The set of psycho-socio-emotional variables was reduced to three PCA components: *Proactivity, Isolation, Inactivity*. The individual degree of risk-aversion toward negative health outcomes was directly related to *Proactivity*, encasing empathic, social support and positive coping strategies, which may prompt individuals to put in place self-protection strategies toward possible negative health consequences. These findings indicate that a risk-averse profile toward possible negative health outcomes may be associated to higher levels of individual prosocial and proactive dispositions, possibly making individuals' more compliant with the social and hygienic guidelines and, thus, reducing their exposure to the SARS-CoV-2 infection.

## Introduction

Italy was one of the first countries in the world, and the very first Western country, to be severely affected by the SARS-CoV2 virus, starting from February 2020. The first pandemic wave, which caused a rapid increase of positive cases and deaths in a few weeks, slowed down. However, in October 2020 the contagion curve raised again (https://www.epicentro.iss.it/coronavirus/sars-cov-2-dashboard) and the Covid-19 pandemic is still causing thousands of deaths worldwide every day (https://covid19.who.int/; https://covid.cdc.gov/covid-data-tracker/#demographics).

After the first lockdown (Mar-May, 2020), the incidence of psychiatric syndromes and psychosocial distress increased significantly in Italy (Sani et al., 2020), as well as in all other countries (Serafini et al., 2020; Torales et al., 2020). In addition, such a new growth of contagions further boosts people's experience of anxiety and distress (Mazza et al., 2020). In this unpredictable context, interindividual differences in risk-taking attitudes – reflecting the subjective willingness to take risks - might represent a crucial variable capable to modulate decision-making and risk-taking behaviors toward negative health outcomes, which also concern the individuals' compliance with Covid-19 containment measures (e.g., social distancing, fiduciary isolation, mask use).

The literature about risk-taking attitude and behaviors in relation to life-threatening events indicates that first-person experience of extremely stressful events can change risk attitudes by either decreasing or increasing individual risk tolerance, namely making people have more risk-averse (Holt and Laury, 2002; Shupp et al., 2017; Jakiela and Ozier, 2018) or risk-prone (Orri Stefánsson and Bradley, 2019; Galandra et al., 2020) attitudes. However, previous studies on this topic have often used tasks including hypothetical monetary stimuli (i.e., simulations of monetary rewards, opposed to real monetary stimuli which allow the subjects to gain or lose a real payoff) (Xu et al., 2016), preventing conclusions about real life contexts and decisions, as well as people's choices in relation to non-monetary outcomes.

Recently, we developed the *Covid-19 Risk Task* (Galandra et al., 2020), starting from the Holt-Laury Paired Lottery Task (Holt and Laury, 2002), including novel ecological stimuli beside the standard monetary lotteries.

Briefly, the Holt-Laury Paired Lottery Task is a classical decision-making task, widely used to eliciting risk preferences and attitudes. It is based on a multiple prize list (MPL) design in which the subject is asked to consider a list of 10 ordered paired lotteries, A and B – i.e., a series of consecutive choices between two outcomes – reported on ten different rows in a table, and indicate which, between lotteries A and B, the subject would accept for each row. In any row, Lottery A represents a safer choice than Lottery B, as the expected payoff of the latter increases at a higher rate than the former. The row at which subjects switch from the safe to the risky lottery (i.e., the switch from A to B) is thus used as a proxy of risk aversion (see details in Materials and Methods section).

Starting from this design, the two ecological versions (*Health Status* and *Employment Status conditions*) of the *Covid-19 Risk Task* (Galandra et al., 2020) were specifically related to risk-taking attitudes toward different real-life domains, concerning health and employment outcomes in the Covid-19 pandemic time. Results highlighted that individuals are more prone to undertake risky behaviors when presented with ecological stimuli (e.g., choosing between two different medical or employment conditions), rather than hypothetical monetary materials (i.e., choosing between two different lotteries) (Galandra et al., 2020). These findings underlined that, when facing ecological stimuli related to a real emergency situation, peoples' decisions for non-monetary outcomes are similar to decisions undertaken in presence of real monetary lotteries putting real payoffs at stake (Xu et al., 2016), and producing a larger shift in risk-taking attitudes (Galandra et al., 2020). Briefly, triggers of real-life experiences as stimuli, also in non-monetary domains, appear more effective to investigate realistic risk-related behaviors, and facilitate the interpretation and contextualization of results.

It is well-known that psychosocial and emotional factors (e.g., loneliness, empathy, coping style, anxiety and mood alterations) influence our perceptions of events (Galandra et al., 2020; Serafini et al., 2020), and represent crucial determinants in risky decision-making (Charpentier et al., 2017; Zhu and Wang, 2017; Taylor, 2020) especially in extremely stressful and life-threatening situations (Brooks et al., 2018; Safi-Keykaleh et al., 2020). Into the context of Covid-19 pandemic, we showed that the perception of the outbreak impact for health could be modulated by the degree of loneliness and distress (Cerami et al., 2020b), as well as by proactive and prosocial attitudes, including empathy, social support and positive coping strategies (Cerami et al., 2020a). Additionally, age may as well have a role in modulating risk-attitude toward negative health outcomes, as young people might perceive themselves having better chances to rapidly recover from Covid-19 or not having severe long-term consequences.

In light of these considerations, we explored the relationship linking individual risk-taking attitude toward health to psycho-socio-emotional dimensions modulating the experience of life-threatening situations and age, in the context of the Covid-19 pandemic. To this purpose, we hypothesized that a more risk-averse attitude toward possible negative health outcomes may be related to superior prosocial dispositions and proactive coping styles, enhancing people's readiness to actively put in place self-protection strategies to cope with such a long-term stressful and health-threatening situation, like the Covid-19 pandemic.

## Materials and Methods

### Participants

The present study included 130 volunteers (89 females, mean age = 38.5 y.o., sd = ±9.3 y.o.) from the general population, who took part to the PsyCOVID Study [https://wprn.org/item/428452; (Cerami et al., 2020b)] and additionally completed the Covid-19 Risk Task (https://psyarxiv.com/5n942/). While the aim of the PsyCOVID Study was collecting multidimensional data, including health status and psycho-socio-emotional variables in Italian residents, the purpose of Covid-19 Risk Task survey was to delineate specific profiles of risk-taking behaviors in working adults (age range = 25–64 y.o.). Both the PsyCOVID Study (Cerami et al., 2020b) and the Covid-19 Risk Task (Galandra et al., 2020) surveys have been implemented on Google Forms and distributed via written invitations through e-mails and Whatsapp.

At the beginning of the survey, we presented the general aim of the study, the commitment required to participants, and information about the research team. Participants had to read and provide their informed consent by clicking a box. After providing informed consent, participants were directed to the survey. Participants did not receive any incentive to take part in the study. Eligibility criteria were the age (18 y.o. or older), the ability to provide an informed consent and the place of residence (Italy).

All participants provided their consent to the experimental procedure, which was approved by the IUSS-University of Pavia Ethics Committee.

### Measures

#### Risk-Taking Attitude Toward Health

Risk-taking attitude toward health was estimated as a result of the *Health Status condition (HSc)* of the Covid-19 Risk Task (Galandra et al., 2020). The *HSc Covid-19 Risk Task* represents a modified version of the classical Holt-Laury Paired Lottery Task (Holt and Laury, 2002) and was specifically developed to assess risk-taking attitude toward negative health outcomes, in the context of Covid-19 pandemic. Briefly, it includes two series of 10 paired Lotteries, A and B, presented on 10 consecutively rows in a table. In any row, Lottery A and Lottery B reflect different health outcomes (*Series 1* Lottery A: Symptomatic SARS-CoV2 infection without hospitalization – Type II Diabetes Mellitus, Lottery B: Shoulder Fracture – Symptomatic SARS-CoV2 infection with hospitalization; *Series 2* Lottery A: Psoriasis – Asymptomatic SARS-CoV2 infection, Lottery B: Cold – Symptomatic SARS-CoV2 infection without hospitalization) [see Supplementary Tables 1, 2 and Galandra et al. for further details about stimuli selection and stimuli appearance (Galandra et al., 2020)].

In this task, participants have to make a choice between Lottery A and Lottery B. In any row, Lottery A always reports the “safe” choice while Lottery B represents the “risky” choice, as Lottery A has less payoffs variability than Lottery B. The 10 rows differed in terms of probability of “winning the higher prize” – i.e., the probability to undergo the less severe negative outcome in terms of health care – in each lottery. In the first row, the probability of winning the higher prize is 10%, while for the subsequent nine rows, the probability to obtain the better outcome progressively increases by 10% so that by row nine there is a 90% chance of winning the higher prize, and row 10 is a choice between two certain winnings.

A *risk-neutral* individual usually selects Lottery A for the first four choices, either A or B for choice five (i.e., 50–50%) and then switches over Lottery B for the last four choices. Considering the utility function

$$\begin{array}{l} \text{u}\left(\text{x}\right)=\text{x}{\;}^{\text{r}-1} \end{array}$$

where x represents the prize and r represents the constant relative risk aversion coefficient (CRRA) (Holt and Laury, 2002; Albert and Duffy, 2012), risk-neutral conditions are defined by *r* = 0, while risk-loving and risk-averse conditions by, respectively, *r* > 0 and *r* < 0. In the present work, we characterized the individual risk-taking profile on the basis of Albert's r cut-offs (Albert and Duffy, 2012) and, thus, we identified participants' as *risk-loving* (*r* < −0.15, n. of safe choices: 0–3), *risk-neutral* (−0.15 < *r* > 0.15, n. of safe choices: 4), *mildly risk-averse* (0.15 < *r* > 0.68, n. of safe choices: 5–6) and *highly risk-averse* (*r* > 0.68, n. of safe choices: >6).

#### Psycho-Socio-Emotional Dimensions

In the PsyCOVID study, we collected a set of measures reflecting psycho-socio-emotional dimensions, with a battery of validated questionnaires in Italian language. In particular, we used the Empathic Concern and Perspective Taking sub-scales of the Interpersonal Reactivity Index – IRI (Davis, 1983) to describe, respectively, emotional and cognitive dimensions of empathic abilities. Loneliness was investigated with the Italian Loneliness Scale – ILS (Zammuner, 2008), which includes three sub-scales: Emotional, Social and General Loneliness. Coping strategies were assessed with the short version of the Coping Orientation to the Problems Experienced – COPE-NVI-25 (Foà et al., 2015), measuring different coping styles toward problems and stressful events, reflected in 5 scale sub-scores (Positive attitude, Problem orientation, Transcendence orientation, Social support, Avoidance strategies). Finally, we collected information about individuals' ability to identify and describe emotions experienced by one's self or others with the Toronto Alexithymia Scale – TAS-20 (Bressi et al., 1996).

### Statistical Analyses

We performed statistical data analysis using SPSS (https://www.spss.it/) and set statistical significance at *p* < 0.05 for all tests.

First, we carried out descriptive statistics on: (i) socio-demographic variables, reporting mean and standard deviation for pseudo-continuous measures and frequency and percentage for categorical descriptors, and (ii) risk-taking attitudes toward health, reporting frequency and percentage of different risk profiles (risk-loving, risk-neutral, mildly risk-averse, highly risk-averse). To this purpose, we computed a measure of risk-taking attitude toward health (*mHSc*) as the mean of number of safe choices between Series 1 and 2.

Then, we performed a Principal Component Analysis (PCA) on psycho-socio-emotional variables in order to identify a smaller set of predictors. In particular, after assessing the suitability of the correlation matrix (Keiser-Meyer-Olkin Measure of Sampling Adequacy = 0.661; Bartlett's test of sphericity < 0.001), we performed a PCA on the scores of 11 variables, including the three sub-scales of the ILS (General Loneliness, Emotional Loneliness, Social Support), the IRI perspective-taking and emotional concern sub-scores, the five scores of coping styles assessed with the COPE-NVI-25 (Positive attitude, Problem orientation, Transcendence orientation, Social support, Avoidance strategies) and the global score of the TAS-20. Both the scree plot and the Kaiser-Guttman criterion (i.e., components with eigenvalue >1) converged in determining the number of components to be retained (=3). We used an orthogonal rotation (Varimax) to facilitate the interpretation of the resulting components (Abdi and Williams, 2010).

We then explored the relationship linking the loading factors of the three independent components to the measure of risk-taking attitude toward health (*mHSc*). We finally assessed the relationship between *mHSc* and age.

## Results

Tables 1, 2 illustrate, respectively, the socio-demographic characteristics of study sample (*n* = 130) and the relative distribution of risk-taking profiles toward health.

**Table 1:** Descriptive characteristics of the sample.

**Sample description**	
Female/Male%	68.5/31.5
Age in years (mean ± sd)	38.5 ± 9.3
Education in years (mean ±sd)	17.3 ± 1.4
Geographical area (Northern Italy/Southern-Central Italy) %	73.8/26.2
Employment condition (employee/freelance) %	63.8/36.2

*The table reports socio-demographic characteristics of study participants*.

**Table 2:** Risk-taking profiles toward health.

**N^**°**^ of safe choices**	**CRRA range**	**Risk-taking attitude classification**	**Risk-taking profile distribution**
0–3	−0.95 < *r* > −0.15	Risk-loving	51.6%
4	−0.15 < *r* > 0.15	Risk-neutral	14.6%
5–6	0.15 < *r* > 0.68	Mildly risk-averse	24.6%
7–10	*r* > 0.68	Highly risk-averse	9.2%

*The table reports information about the distribution of risk-taking profiles toward health in the study sample (n = 130)*.

As reported in Table 2, about half of the sample (67/130, 51.6%) showed a risk-loving profile, while 19 out of 130 subjects showed a risk-neutral profile and a third of the sample 44/130 included mildly to highly risk-averse individuals.

PCA reduced the initial dataset of 11 psycho-socio-emotional variables into 3 components explaining the 59.317% of the total variance. This result was in line with findings reported in the overall sample of the PsyCOVID study, including a total number of 1,258 participants (Cerami et al., 2020b). The first component (C1: *Proactivity*) included active, problem oriented and social support coping strategies, plus variables related to empathy, suggesting an internal locus of control. The second component (C2: *Isolation*) encompassed the two loneliness scores. Finally, the third component (C3: *Inactivity*) suggested an external locus of control, with transcendent and avoidant coping strategies, alexithymia and social loneliness sub-score.

To explore the relationship between C1, C2 and C3 to the measure of risk-taking attitude toward health (*mHSc*), we computed a correlation analysis (Spearman's rank correlation coefficient), which revealed a significant positive relationship between *mHSc* and C1 (*r* = 0.25, *p* = 0.005). No significant correlation was found between *mHSc* and the other two components C2 (=-0.5, *p* = 0.601) and C3 (=-0.1, *p* = 0.270). This result suggests that the increase of risk-aversion toward possible negative health outcomes is related to prosocial and proactive dispositions reflecting an internal locus of control.

Finally, the correlation analysis (Spearman's rank correlation coefficient) between *mHSc* and age highlighted a positive significant association (*r* = 0.2, *p* = 0.026) indicating that the risk aversion toward negative health outcomes increase with age.

## Discussion

Covid-19 pandemic is putting the whole society to the test. Social distancing, fear of contagion and job uncertainty became part of our lives. In such an unpredictable and stressful situation, personal resources needed to promote psychosocial adaptation and emotional balance may be lacking, and this in turn may affect routines and habits related to everyday life and work (Cellini et al., 2020; León-Zarceño et al., 2021). In particular, into the context of Covid-19 pandemic, the risk of falling ill is weighed against the risk of losing the job and, thus, possibly compromising the living standards (Godinic et al., 2020; Rutter et al., 2020). In addition, people who work, find themselves having to organize and manage job activities and childcare as best as possible in this uncertain situation, sometimes without sufficient support of the institutions, trying to appropriately balance needs and sustainability (Del Boca et al., 2020a,b; Leduc and Liu, 2020; Blum and Dobrotić, 2021; Ruffolo et al., 2021).

As we reported by analyzing the baseline findings of the PsyCOVID study (Cerami et al., 2020b), loneliness and distress, but also empathic skills and proactive coping strategies, represent psychosocial and emotional determinants shaping individual judgments and perceptions, as well as risky decision-making processes (Charpentier et al., 2017; Zhu and Wang, 2017; Taylor, 2020).

Investigating interindividual differences in risk-taking attitudes toward negative health outcomes through an *ad-hoc* developed risk task – *Covid-19 Risk* Task (Galandra et al., 2020) in 130 Italian workers (89 females, age range 25–64) collected among the PsyCOVID study participants, we found an opposite pattern of risk-taking profiles in health *vs*. monetary condition with more frequent risk-loving behaviors in playing health-related lotteries. Thus, we decided to further explore and report in the present work the relationship between individual differences in risk-taking attitude toward health and psycho-socio-emotional variables modulating the individual experience during life-threatening situations, and in turn people's resilience to Covid-19 pandemic.

In detail, half sample showed a risk-loving attitude toward negative health outcomes in the *HSc* condition of the *Covid-19 Risk Task* despite the greatest part maintained a risk-averse profile in the monetary condition [mildly to highly risk-averse 108/130 (83.1%); risk-neutral 11/130 (8.5%); risk-lovers 11/130 (8.5%)] (Galandra et al., 2020). This evidence further confirmed the shift in risk-taking attitude and behaviors when people are facing or have recently faced extremely stressful conditions, as underlined in previous studies (Brooks et al., 2018; Cerami et al., 2020b). In this case, people were more prone to undertake a risky decision – i.e., half of participants showed a risk-loving profile – when they had to choose between different medical conditions, including the risk to develop Covid-19 symptomatology. Moreover, young compared to older adults might perceive themselves as less vulnerable to the infection or having better chances to recover from Covid-19. For these reasons they might be more willing to undertake risky decisions for their health in order to obtain the best possible outcome. Our results support this hypothesis by showing that the increase of risk aversion toward negative health outcome is positively associated with age. Similar results have been previously provided in adolescents and young adults that perceived themselves less at risk of infection compared to their relatives (Yang et al., 2020) and took the pandemic less seriously (Commodari and La Rosa, 2020). Consistently, young adults with higher risk perception reported stronger desire to contribute in the reduction of contagion and to protect their loved ones compared to peers with lower risk perception (Yang et al., 2020).

**Figure 1:**
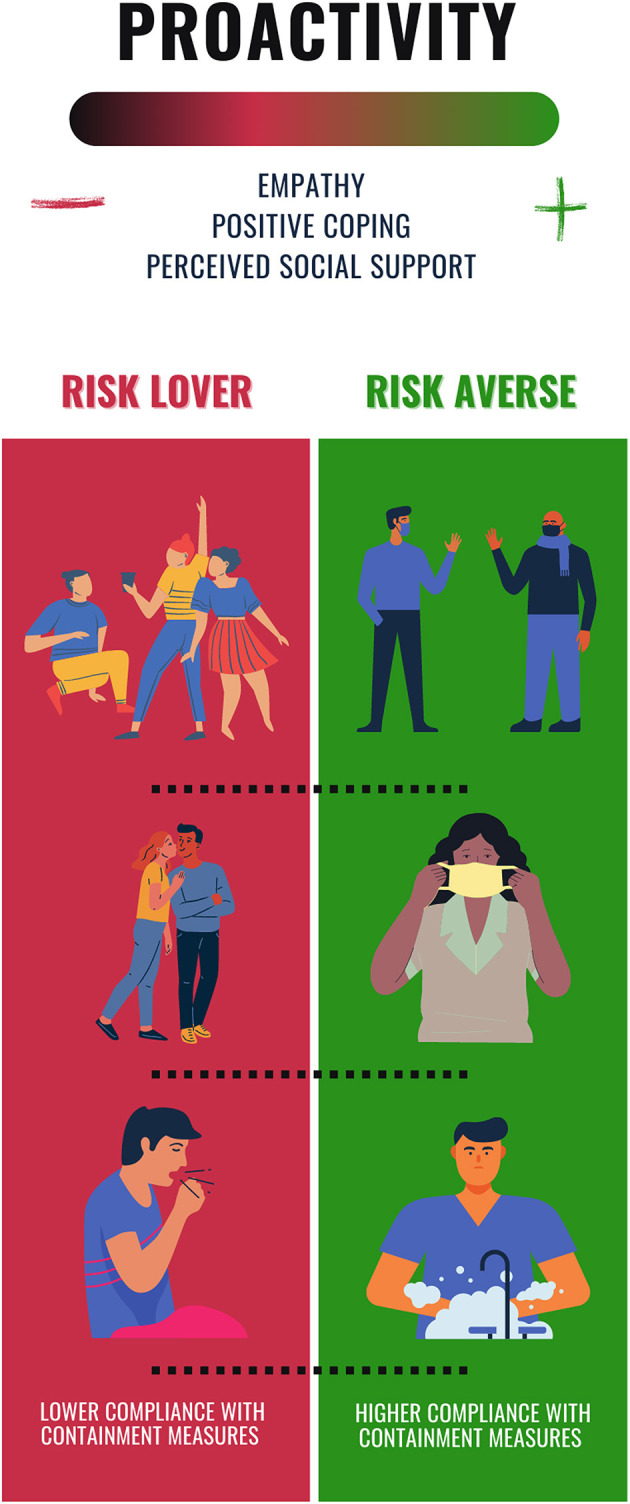
Proactivity, risk-taking attitude and compliance with containment measures. The figure illustrates the relationship between *Proactivity* and risk-taking attitude toward negative health outcomes, and the possible consequences in the individual compliance with infection containment measures, in the context of Covid-19 pandemic.

In line with recent findings (Commodari et al., 2020), that highlighted the role of psychological variables such as empathy, self-efficacy and imagination in promoting an overall healthy behavior and a better compliance with Covid-19 containment measures, we provided evidence that people's risk-taking profile toward health is related to individual differences in psycho-socio-emotional variables. Indeed, our data revealed that the individual degree of aversion toward risk for health is positively related to a PCA component – i.e., *Proactivity* – encasing proactive, problem oriented and social support coping strategies, plus superior empathic skills (Figure 1).

To put it differently, we observed that people with a risk-loving profile toward health (51.6% of our sample) showed an inferior degree of *Proactivity* than more risk-averse individuals, and thus displayed a lower expression of empathic concern and perspective-taking, a less use of positive coping styles, and lower degrees of perceived social support. These dimensions have been related to the health locus of control, which impact on how people approach their own health and health-related life decisions (Kesavayuth et al., 2020). Moreover, a higher risk tolerance toward health problems – which is conceptually similar to the risk-loving attitude assessed by the health condition of the *Covid-19 Risk Task* – has been associated to *chance health locus of control* (Wallston et al., 1978), namely believing that an external force (e.g., the fate) governs our health status.

Individuals showing a greater risk-aversion toward possible negative health outcomes revealed superior prosocial dispositions and perceived social support, as well as positive coping styles might be characterized by and internal health locus of control (i.e., believing that there is a direct link between one's behavior and health status) which may enhance individuals' readiness to actively put in place self-protection behaviors (e.g., social distancing, mask use, hand hygiene) to cope with the distress and the threat that Covid-19 pandemic posed on our lives for an indefinite period of time. Adopting a positive coping style encourages to better assess information coming from the environment, reducing anxious, fearful and depressive feelings to stressful condition and finally promoting adherence to regulations and directives (Ding et al., 2020).

Major limitations to the present work of course refer to the lack of a longitudinal perspective and the adoption of a small sample size. Indeed, the cross-sectional nature of the study design prevents any kind of causal conclusion about possible changes of individual risk-taking attitudes as a consequence of life-threatening and stressful experiences, like that of the Covid-19 pandemic, overtime. In addition, the small sample size and the selective age range may hinder the generalization of these findings to the general population. Thus, only future replication studies, using same tasks on larger samples and including younger (<18 y.o.) and older (>65 y.o.) individuals, will be able to confirm the reliability of present results and overcome limitations of the cross-sectional study design. Importantly, despite we found an association between risk-taking attitudes toward negative health outcomes and proactivity suggesting a relationship with individual compliance to regulations aimed at containing the pandemic spread, our findings are not sufficient to explain individual behaviors put in place and compliance to government directives. Further studies specifically exploring risk-attitude profiles and compliance to hygienic and social recommendations are recommended.

In conclusion, the present study highlights how shifts in risk-taking attitudes by preferring possible negative health outcomes are related to the psychological and socio-emotional individual profile. This is of extreme importance in the context of the present Covid-19 pandemic in which individual behaviors may dramatically influence the well-being of the whole community. Excessive risk tolerance toward negative health outcomes together with the believe that individual actions and compliance to social and hygienic guidelines – e.g., respecting the social distancing, wearing the mask, or washing hands properly – are not necessarily linked to negative health consequences may cause the whole community to be more exposed to the SARS-CoV2 diffusion. Since the psycho-socio-emotional profile in risk-loving people is characterized by a lower degree of empathic dispositions and perceived social support, beside a scarce use of positive coping strategies, novel and multi-domain intervention strategies should be developed to overcome the psychosocial crisis that is spreading all over the world. Such interventions should promote positive attitude and resilience to the crisis and self-efficacy in adhering to the restrictive measures to contain virus contagion. Specific interventions including psychoeducational and metacognitive approaches, as well as mindfulness trainings, may also help to increase self-awareness and improve the empowerment of empathic and social skills in order to reduce emotional distress and perceived isolation and boost social support in individuals in daily life and crisis times. In the meantime, the scientific community should be better aware of the psychosocial impact that the Covid-19 pandemic is going to have to Western and Eastern populations (AlHumaid et al., 2020; Chew et al., 2020; Dawson and Golijani-Moghaddam, 2020; Rodríguez-Rey et al., 2020; Xiong et al., 2020). Parallelly, governments should consider the need of allocating the available economic resources to large-scale psychological interventions, with the aim to increase people's resilience according to the needs of psychosocial well-being in the whole society and the specific requirements of some fragile populations.

## Data Availability Statement

The raw data supporting the conclusions of this article will be made available by the authors, without undue reservation.

## Ethics Statement

The studies involving human participants were reviewed and approved by IUSS-University of Pavia Ethics Committee. The patients/participants provided their written informed consent to participate in this study.

## Author Contributions

CG, CCe, and CCr participated in study design and performed data analysis and interpretation. CCe and CCr drafted the main manuscript. All authors participated in data collection and reviewed the manuscript.

## Conflict of Interest

The authors declare that the research was conducted in the absence of any commercial or financial relationships that could be construed as a potential conflict of interest.
