# Supplementary material for: Risk-Aversion for Negative Health Outcomes May Promote Individual Compliance to Containment Measures in Covid-19 Pandemic
Source: Front Psychol. 2021 Jun 18;12:666454. doi: 10.3389/fpsyg.2021.666454 (PMC8249698; doi:10.3389/fpsyg.2021.666454)
Supplement: Supplementary file 1 [file Table_1.DOCX]

**SUPPLEMENTARY INFORMATION**

**Risk-aversion for negative health outcomes may promote individual compliance to containment measures in Covid-19 pandemic**

*Authors*

Chiara Cerami, Caterina Galandra, Gaia C Santi, Alessandra Dodich, Stefano F Cappa, Tomaso Vecchi, Chiara Crespi

**Table S1 – Health status Condition development.**

The table reports a list of 24 items corresponding to different medical conditions we used to create the Health status Condition (HsC) of the Covid-19 Risk Task. In order to select the final HsC items, we asked 38 healthy subjects (age range: 20-70 y.o.) to rate a larger set of items in terms of perceived illness severity on a 5-point Likert scale (*0=not severe at all; 1=Not very severe; 2= Severe enough; 3=Severe; 4=Extremely severe*). We then sorted the items on the basis of the average severity perception (Columns B-C) and selected those having the same constant relative risk aversion (CRRA) coefficient of the items included in the monetary condition derived by the Holt-Laury Paired Lottery Task.

| **A: Items** | **B: Rating**  **(average data)** | **C: Raking of**  **Severity perception** |
| --- | --- | --- |
| Cold | 1.21 | 1 |
| Sore throat | 1.50 | 2 |
| Cough | 1.71 | 3 |
| Diarrhea | 1.76 | 4 |
| Hemorrhoids | 1.76 | 5 |
| Headache | 1.92 | 6 |
| Gastroesophageal reflux | 2.08 | 7 |
| Cystitis | 2.13 | 8 |
| Allergy | 2.21 | 9 |
| Psoriasis | 2.24 | 10 |
| Shoulder fracture | 2.42 | 11 |
| Asymptomatic Covid-19 infection | 2.63 | 12 |
| Arthrosis | 2.82 | 13 |
| Bronchial asthma | 2.84 | 14 |
| Osteoporosis | 2.95 | 15 |
| Thighbone fracture | 3 | 16 |
| Symptomatic Covid-19 infection without hospitalization | 3 | 17 |
| Diabetes mellitus | 3.53 | 18 |
| Symptomatic Covid-19 infection with hospitalization | 4.05 | 19 |
| AIDS | 4.37 | 20 |
| Alzheimer’s disease | 4.37 | 21 |
| Lung cancer | 4.45 | 22 |
| Heart attack | 4.47 | 23 |
| Stroke | 4.53 | 24 |
