# Supplementary material for: Risk-Aversion for Negative Health Outcomes May Promote Individual Compliance to Containment Measures in Covid-19 Pandemic
Source: Front Psychol. 2021 Jun 18;12:666454. doi: 10.3389/fpsyg.2021.666454 (PMC8249698; doi:10.3389/fpsyg.2021.666454)
Supplement: Supplementary file 2 [file Table_2.DOCX]

**SUPPLEMENTARY INFORMATION**

**Risk-aversion for negative health outcomes may promote individual compliance to containment measures in Covid-19 pandemic**

*Authors*

Chiara Cerami, Caterina Galandra, Gaia C Santi, Alessandra Dodich, Stefano F Cappa, Tomaso Vecchi, Chiara Crespi

**Table S2 – Covid-19 Risk Task Health Status Condition, Series 1**

The table reports 10 paired lotteries (Lottery A and Lottery B) included in the Series 1 of the Covid-19 Risk Task Health Status Condition. Stimuli in the paired lotteries are represented by negative health outcomes.

| *Covid-19 risk task health status condition* | | | |
| --- | --- | --- | --- |
| **Rows** | **Lottery A** | **Lottery B** | **Relative risk aversion coefficient (CRRA)** |
| 1 | 10% Symptomatic Covid-19 infection without hospitalization—90% Type II Diabetes Mellitus | 10% Shoulder Fracture—90% Symptomatic Covid-19 infection with hospitalization | r<−0.95 |
| 2 | 20% Symptomatic Covid-19 infection without hospitalization—80% Type II Diabetes Mellitus | 20% Shoulder Fracture—80% Symptomatic Covid-19 infection with hospitalization | −0.95<r>−0.49 |
| 3 | 30% Symptomatic Covid-19 infection without hospitalization—70% Type II Diabetes Mellitus | 30% Shoulder Fracture—70% Symptomatic Covid-19 infection with hospitalization | −0.49<r>−0.15 |
| 4 | 40% Symptomatic Covid-19 infection without hospitalization—60% Type II Diabetes Mellitus | 40% Shoulder Fracture—60% Symptomatic Covid-19 infection with hospitalization | −0.15<r>0.15 |
| 5 | 50% Symptomatic Covid-19 infection without hospitalization—50% Type II Diabetes Mellitus | 50% Shoulder Fracture—50% Symptomatic Covid-19 infection with hospitalization | 0.15<r > 0.41 |
| 6 | 60% Symptomatic Covid-19 infection without hospitalization—40% Type II Diabetes Mellitus | 60% Shoulder Fracture—40% Symptomatic Covid-19 infection with hospitalization | 0.41<r > 0.68 |
| 7 | 70% Symptomatic Covid-19 infection without hospitalization—30% Type II Diabetes Mellitus | 70% Shoulder Fracture—50% Symptomatic Covid-19 infection with hospitalization | 0.68<r > 0.97 |
| 8 | 80% Symptomatic Covid-19 infection without hospitalization—20% Type II Diabetes Mellitus | 80% Shoulder Fracture—20% Symptomatic Covid-19 infection with hospitalization | 0.97<r > 1.37 |
| 9 | 90% Symptomatic Covid-19 infection without hospitalization—10% Type II Diabetes Mellitus | 90% Shoulder Fracture—10% Symptomatic Covid-19 infection with hospitalization | r>1.37 r>1.37 |
| 10 | 100% Symptomatic Covid-19 infection without hospitalization—0% Type II Diabetes Mellitus | 100% Shoulder Fracture—0% Symptomatic Covid-19 infection with hospitalization | r<−0.95 |
